# New Coumarin Derivatives as Cholinergic and Cannabinoid System Modulators

**DOI:** 10.3390/molecules26113254

**Published:** 2021-05-28

**Authors:** Serena Montanari, Marco Allarà, Laura Scalvini, Magdalena Kostrzewa, Federica Belluti, Silvia Gobbi, Marina Naldi, Silvia Rivara, Manuela Bartolini, Alessia Ligresti, Alessandra Bisi, Angela Rampa

**Affiliations:** 1 Department of Pharmacy and Biotechnology, Alma Mater Studiorum University of Bologna, Via Belmeloro 6, 40126 Bologna, Italy; serena.montanari5@unibo.it (S.M.); federica.belluti@unibo.it (F.B.); silvia.gobbi@unibo.it (S.G.); marina.naldi@unibo.it (M.N.); manuela.bartolini3@unibo.it (M.B.); 2 Endocannabinoid Research Group, Institute of Biomolecular Chemistry, National Research Council, Via Campi Flegrei 34, 80078 Pozzuoli, Italy; mallara@icb.cnr.it (M.A.); m.kostrzewa@icb.cnr.it (M.K.); aligresti@icb.cnr.it (A.L.); 3 Department of Pharmacy, University of Parma, Parco Area delle Scienze 27/A, 43124 Parma, Italy; laura.scalvini@unipr.it (L.S.); silvia.rivara@unipr.it (S.R.)

**Keywords:** Alzheimer’s disease, AChE, BuChE, FAAH, CB receptors, Aβ_42_ self-aggregation, carbamate, amide

## Abstract

In the last years, the connection between the endocannabinoid system (eCS) and neuroprotection has been discovered, and evidence indicates that eCS signaling is involved in the regulation of cognitive processes and in the pathophysiology of Alzheimer’s disease (AD). Accordingly, pharmacotherapy targeting eCS could represent a valuable contribution in fighting a multifaceted disease such as AD, opening a new perspective for the development of active agents with multitarget potential. In this paper, a series of coumarin-based carbamic and amide derivatives were designed and synthesized as multipotent compounds acting on cholinergic system and eCS-related targets. Indeed, they were tested with appropriate enzymatic assays on acetyl and butyryl-cholinesterases and on fatty acid amide hydrolase (FAAH), and also evaluated as cannabinoid receptor (CB1 and CB2) ligands. Moreover, their ability to reduce the self-aggregation of beta amyloid protein (Aβ_42_) was assessed. Compounds **2** and **3**, bearing a carbamate function, emerged as promising inhibitors of hAChE, hBuChE, FAAH and Aβ_42_ self-aggregation, albeit with moderate potencies, while the amide **6** also appears a promising CB1/CB2 receptors ligand. These data prove for the new compounds an encouraging multitarget profile, deserving further evaluation.

## 1. Introduction

Alzheimer’s disease (AD), currently considered the most common form of dementia, is a fatal illness inducing a progressive decline in memory and in other aspects of cognition. The complexity of its onset and progression poses one of the most difficult medical challenges of our time [1]. The risk of developing AD is influenced by both genetic and environmental factors; however, the main risk factor is by far age. As a consequence, the current aging population trend makes the disease prevalence more likely to rise [2]. Three classical neuropathological changes in the brains of patients typically define AD: the accumulation of extracellular aggregated β-amyloid protein (to form senile plaques) and intracellular aggregation of hyperphosphorylated tau protein (to form neurofibrillary tangles), together with a severe loss of cholinergic innervation in the cerebral cortex [3]. Indeed, a marked reduction of acetylcholine (ACh), causing a loss of cholinergic tone, is responsible for the severe cognitive impairment characteristic of AD: the “cholinergic hypothesis”, formulated in 1982, was then the first and most studied approach that described AD pathophysiology [4]. In particular, the inhibition of cholinesterases (ChEs), the enzymes catalyzing the degradation of ACh, remains to date the most successful approach for the symptomatic treatment of the disease. In human brain, two main forms of ChEs are involved in ACh hydrolysis, namely acetylcholinesterase (AChE) and butyrylcholinesterase (BuChE). In healthy brain, AChE is the principal responsible for ACh breakdown, while in AD-affected brain AChE levels decline, and BuChE activity progressively becomes prevailing. Consequently, in AD patients, the regulation of central ACh levels relies on the activity of both enzymes.

Currently, three drugs approved by FDA act at a synaptic level by inhibiting ChEs [5], and despite the limited clinical outcomes, research on the central cholinergic system is still thriving. Indeed, recent studies suggest that long-term use of these drugs may also have disease-modifying benefits [6]. Although the precise mechanisms of cholinergic deficit in AD remain to be deeply elucidated, a growing body of evidence suggests that complex reciprocal interactions exist between central cholinergic changes and other pathophysiological features of AD, including abnormal Aβ protein, tau cascade and inflammation [7]. A better elucidation of these interconnections could contribute to a reconsideration of the cholinergic hypothesis, leading to a proper targeting of the cholinergic system, which may also be involved in modifying AD disease progression [8]. In particular, BuChE has been found to be responsible for up-regulating the expression of the amyloid precursor protein (APP) in cell membranes which undergoes cleavage by β- and γ-secretase enzymes leading to Aβ production in a process called the amyloidogenic pathway. Thereby, inhibition of BuChE could be regarded as an additional approach for the treatment of moderate forms of AD, as it would result in the increase of ACh synaptic levels and the decrease of neurotoxic Aβ fibrils [9].

The term “endocannabinoid system” (ECS) refers to the two cannabinoid receptors CB1 and CB2, the endocannabinoids (eCBs), and a group of enzymes involved in their synthesis and breakdown. eCBs constitute a relatively small group of fatty acid-derived endogenous ligands of CB1 and CB2 receptors [10]. The first two eCBs discovered, anandamide (*N*-arachidonoylethanolamine, AEA) and 2-arachidonoylglycerol (2-AG), are still the most studied. In neurons, eCBs are synthesized and released “on demand”, in several physiological and pathophysiological conditions, via different multi-step processes and are catabolized by enzymatic hydrolysis [11]. Fatty acid amide hydrolase (FAAH, now also known as FAAH-1), distributed throughout the brain as an integral membrane protein, is the major catabolic enzyme of AEA. Hence, it has been considered a promising therapeutic target in different inflammatory diseases, pain and other disorders [12]. 2-AG is mainly hydrolyzed via the enzyme monoacylglycerol lipase (MAGL), but also by FAAH [13].

In recent years, the involvement of eCBs in neuroprotection has been assessed, and a number of studies have proven that activation of eCB signaling can suppress microglial activation and hamper the neurodegenerative processes involved in several neurological diseases [14,15]. Accordingly, targeted pharmacotherapeutic manipulations of eCB signaling have been undertaken in order to attain neuroprotective outcomes; in particular, it may be indirectly enhanced to therapeutic levels through FAAH inhibition, making FAAH an attractive target and selective FAAH inhibitors promising drug candidates for various neurological and neurodegenerative/neuroinflammatory disorders, including AD [16]. Indeed, FAAH inhibitors seem more appropriate to exploit the neuroprotective nature of eCB signaling compared to chronic application of direct CB1 agonists, thanks to a lower risk of psychotropic (or other) adverse effects.

The lack of an effective treatment for AD is mainly related to the involvement of multiple and complex pathways in its onset and evolution, making any drug endowed with a single target-mechanism of action essentially ineffective. The polypharmacology approach, i.e., the delivery of a “cocktail” of two or more drugs, could thus be beneficial for treating such complex pathologies, even if poor patient compliance and pharmacokinetic interferences may negatively affect this therapeutic strategy. Alternatively, a single chemical compound could be purposely designed to simultaneously engage different targets and/or pathways involved in the multifactorial nature of the disease. This multitarget approach would greatly enhance the probability of disease modification and would be more appreciated by patients [17,18].

**Figure 1:**
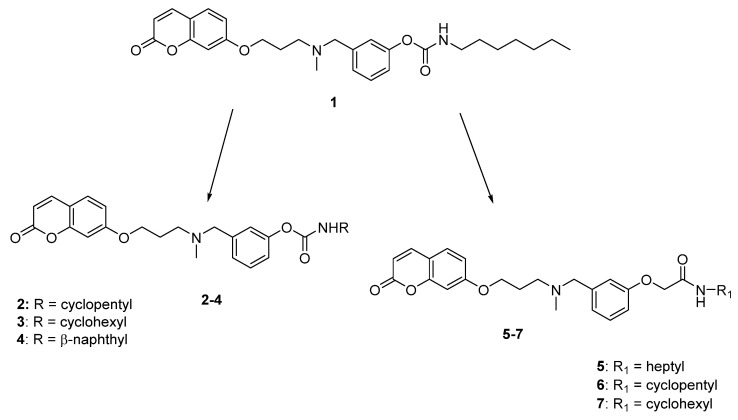
Structure of lead compound **1** and design strategies for the new compounds.

In this respect, we recently reported a series of compounds as first dual ChE/FAAH inhibitors, with activity in the nanomolar range [19,20]. A structure-activity relationship (SAR) study was performed and compound **1** (Figure 1) emerged as one of the most potent BuChE inhibitors reported to date (IC_50_ = 1.36 nM) and as a well-balanced AChE and FAAH inhibitor (IC_50_ = 37.4 nM e 28.5 nM, respectively). From this study, some significant structural data resulted, in particular:

- the coumarin moiety gave the best results as core structure. Notably, this natural-derived scaffold is recognized as “privileged structure” in medicinal chemistry, and it is widely used in drug design [21,22];
- the carbamate function is the key fragment for the interaction with the catalytic sites of ChEs and FAAH where it is supposed to carbamoylate the catalytic serine residue;
- the *N,N’*-benzylmethylamino group gave potent enzyme inhibition and was proposed as the recognition element for the anionic sites of both AChE and BuChE;
- a 3-methylene units linker between the coumarin and the amino function provided high and balanced inhibition activity.

In this paper, appropriately designed modifications were performed on our lead compound **1**, in order to further explore the chemical space of these multi-target inhibitors and outline a more complete picture of their SARs (Figure 1), focusing on the carbamate group. In detail, this portion was modified by enclosing the heptyl chain of **1** in a 5- or 6-membered ring (compounds **2** and **3**, respectively), or by introducing a *N*-naphthyl substituent (compound **4**). These modifications, aimed at maintaining or reducing the considerable lipophilicity of the parent compound, could improve the steric interactions with the binding pocket of the target enzymes; notably, the cyclohexyl carbamate is also present in the selective FAAH inhibitor URB597 [23]. Moreover, a small series of derivatives was designed and synthesized replacing the carbamate with an amide group (compounds **5**–**7**), a favourable moiety widely reported in drugs, intermediates, pharmaceuticals, and natural products [24], due to its ability to form important hydrogen bonding interactions. This substitution aimed at elucidating the role of the carbamate function in enzyme blockade and evaluating the feasibility of attaining reversible inhibitors of ChEs and FAAH.

## 2. Results

### 2.1. Chemistry

**Scheme 1:**
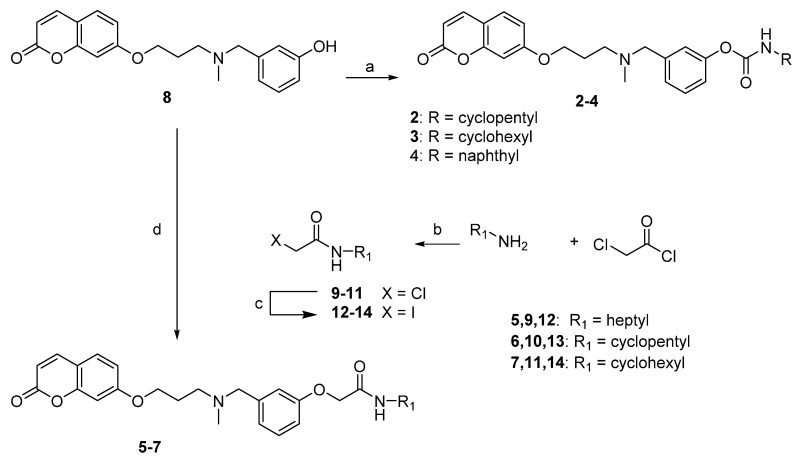
Synthesis of compounds **2–7**. Reagents and conditions: (**a**) RNCO, NaH, toluene, rt, 24 h; (**b**) K_2_CO_3_, acetone, reflux, 10 h; (**c**) NaI, MeCOEt, reflux; 3 h; (**d**) 12–14, K_2_CO_3_, acetone, reflux, 24 h.

The synthesis of compounds **2**–**7** was accomplished as shown in Scheme 1. The starting synthon **8**, synthesized according to our previously described procedure [20], was treated with the selected isocyanate, in the presence of NaH, to give the desired compounds **2**–**4**. Alternatively, **8** underwent an alkylation reaction with the selected 2-iodo-*N*-alkylacetamide **12**–**14**, in the presence of K_2_CO_3_, to obtain compounds **5**–**7**. The 2-iodo-*N*-alkylacetamide intermediates **12**–**14** were prepared by reacting 2-chloroacetyl chloride with the selected amine in the presence of K_2_CO_3_ to obtain amides **9**–**11**, which were then reacted with NaI to obtain the iodinated analogues **12**–**14**, more reactive species for the following Williamson reaction.

### 2.2. Biological Evaluation

#### 2.2.1. Predicted Pharmacokinetic Parameters

Lipophilicity (QPlogPo/w) and solubility (QPlogS) were calculated for compounds **1**–**7** and are reported in Table 1. 

Compared to the reference heptyl derivative **1**, the new compounds **2**–**7** show lower QPLogPo/w, which leads to higher predicted solubility and potential improved in vivo distribution.

#### 2.2.2. Cholinesterases and FAAH Inhibition by Compounds **2–7**

The newly synthesized compounds were evaluated against human recombinant AChE (hAChE) and BuChE from human serum (hBuChE), using the method of Ellman [25] and the inhibitory potencies, expressed as IC_50_ values, are reported in Table 2 (compounds **2**–**4**) and 3 (compounds **5**–**7**). Inhibition values (IC_50_s) for compound **1** and rivastigmine, the only marketed carbamate drug approved for AD treatment, are also listed in Table 2 for comparison.

Regarding carbamates **2**–**4**, some considerations can be drawn. As already mentioned, previously reported SAR studies [20] identified the simple coumarin core as the best nucleus for activity. Therefore, it was retained in the new derivatives. In order to evaluate the effects of lipophilicity and size of the carbamic substituent, the linear heptyl chain of the lead **1** was enclosed in a 5- or 6- membered ring, to obtain **2** and **3**, showing a lower logP value and an increased steric hindrance. In compound **4**, the bulkier and planar naphthyl group was introduced.

**Figure 2:**
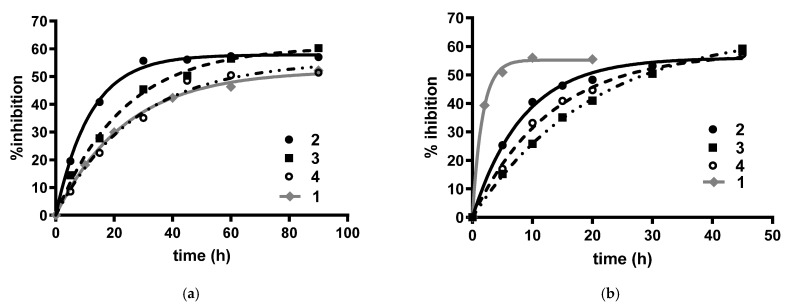
Time-dependent inhibition of (**a**) hAChE and (**b**) hBuChE by carbamates **2–4** (black lines) and lead compound **1** (grey line) at their IC_50_ concentrations.

The inhibition of ChEs by carbamates involves the formation of a reversible complex with subsequent formation of a covalent adduct [26]. It is also well known that carbamoylation rate is influenced by the nature of the substituent at the N-carbamoyl group, as previously shown for **1** [20]. Hence, by bearing different R groups, derivatives **2**–**4** may require different (incubation) time to form the covalent adduct and inhibit AChE and BuChE activities. Given these premises, we initially investigated the time-dependent ChEs inhibition by **2**–**4** by evaluating the time required to achieve a plateau in the inhibition plot. As shown for **1** [20] and other carbamates [19,27], due to the larger enzyme gorge (790 Å vs. 1133 Å [28]) that allows accommodating bulkier substituents, the carbamoylation kinetics was faster on hBuChE than hAChE, independently from the nature of the R substituent (see Figure 2).

The fastest carbamoylation rate was observed in both cholinesterase enzymes with the cyclopentyl-derivative **2**, with the following trends: cyclopentyl- (**2**) > cyclohexyl (**3**) > naphtyl-derivative (**4**), and cyclopentyl- (**2**) > naphtyl (**4**) > cyclohexyl-derivative (**3**) for the carbamoylation of hAChE and hBuChE, respectively. When the carbamoylation kinetics of new derivatives were compared to that previously determined for **1**, it was observed that replacement of the heptyl chain of **1** led to opposite effects on hAChE and hBuChE. More in detail, for AChE inhibition, replacement of the heptyl chain of **1** with a cyclopentyl (**2**) or a cyclohexyl (**3**) group eased the carbamoylation reaction, while no significant difference was observed between **1** and **4**. Conversely, for hBuChE inhibition, all new carbamates showed a significantly slower carbamoylation kinetics compared to that of **1**. Hence, as a consequence of this opposite trend, although a difference still remains, the inactivation rates of the two cholinesterases by **2–4** were, in general, less divergent than those observed for **1**, making an in vivo inhibition of both cholinesterases more likely to happen. As for the last aspect, hAChE carbamoylation by the drug rivastigmine occurs in a time frame close to that observed for **2**, being the pseudo-first order k_obs_ values for rivastigmine and **2**, determined at their IC_50_ value, 0.059 min^−1^ [27] and 0.038 min^−1^, respectively.

Considering that time clearly affects the inhibition rate, in order to achieve a proper comparison of the inhibitory potencies, in the determination of the IC_50_ values the incubation time was adjusted according to the time required to each carbamate to form the covalent adduct, following the same approach previously used for the determination of the inhibitory potency of **1** [20]. This strategy also allowed a better definition of the SARs of this new class of carbamates.

All the tested compounds turned out to inhibit hBuChE more effectively than hAChE, and the potency trend observed was naphthyl > cyclopentyl > cyclohexyl. In particular, going from the cyclopentyl derivative **2** to the homologue cyclohexyl **3**, a four-fold decrease in activity can be observed, whereas the introduction of the planar naphthyl group in derivative **4** allowed obtaining the most potent BuChE inhibitor in the series, with activity in the low nanomolar range and comparable to that of compound **1**. Conversely, these modifications negatively affected the inhibitory activity on hAChE, with potency decreasing from cyclohexyl to naphthyl and cyclopentyl, with the most potent derivative **3** showing a three-fold reduction in potency with respect to **1**. Remarkably, all carbamic compounds proved to be more active than the marketed drug rivastigmine, with compound **3** showing a fifteen-fold increase in potency. The peculiar selectivity profile of this subset of compounds makes them of particular interest in treating moderate forms of AD, where the progressive reduction of AChE levels in the cholinergic synapses upgrades the role of BuChE, which becomes the main responsible for central cholinergic tone regulation.

Looking at the inhibitory activity on FAAH, expressed as IC_50_ values against AEA hydrolysis by FAAH in rat brain membranes and reported in Table 2, a ten-fold reduction in activity can be noticed with respect to **1**. As reported for BuChE, the cycloalkyl carbamates **2** and **3** were the least active, with the cyclohexyl derivative **3** slightly more potent. Surprisingly, these coumarin-based derivatives seem not to benefit from the introduction of cycloalkyl groups in the carbamic function, unlike biphenyl derivatives such as URB597 [23]. Notably, compound **4**, bearing the aromatic naphthyl group, again proved to be the most promising, with potency in the low micromolar range.

A docking study has been performed (see below) to rationalize SARs and to get insights into the molecular interactions with the targets. In this respect, the discrepancy between the IC_50_ values observed with or without the pre-incubation lapse seems to strongly support a covalent inhibition mechanism.

The selectivity towards the other regulator of eCBs levels, MAGL, was also evaluated and all the compounds turned out to be inactive (data not shown). This finding is actually convenient, as dual FAAH/MAGL inhibition was reported to induce cannabimimetic side effects [29].

Taking into account the increasing relevance of amide-containing compounds in medicinal chemistry [24], a small set of easily affordable amide derivatives **5**–**7** were designed and synthesized in order to evaluate the significance of the carbamic group in enzymatic inhibition. As reported in Table 3, the results of biological evaluation clearly indicated a pivotal role for the carbamic function in enzyme inhibition, showing amide derivatives reduced activities on both ChEs and FAAH. 

In particular compound **5**, bearing the same linear heptyl chain of our lead **1**, demonstrated an impressive decrease in inhibitory potency towards hBuChE, while the cyclopentyl and cyclohexyl analogues retained IC_50_ values in the low micromolar range. On hAChE, only compound **6**, bearing the cyclopentyl group, exhibited an interesting inhibitory activity profile. The structural requirements for FAAH inhibition seemed even more limiting, being all amide compounds inactive both on this target and on MAGL (data not shown).

#### 2.2.3. BuChE and FAAH Docking Studies

**Figure 3:**
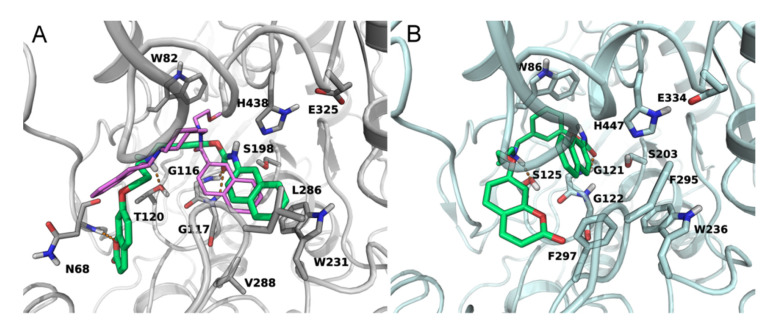
(**A**) Docking of compound 4 (green carbons) in the substrate binding site of *h*BuChE (grey ribbons). The coumarin head points towards the entrance of the gorge undertaking polar contacts with the backbone of N68. The *N*-naphthyl group fits the acyl chain binding pocket, overlapping the position of the naphthamide group of the inhibitor crystallized in the X-ray structure 4TPK (purple carbons). (**B**) Docking of compound 4 (green carbons) in the substrate binding site of *h*AChE (cyan ribbons). Given the presence of the bulkier F295 and F297, the *N*-naphthyl group cannot occupy the acyl chain binding pocket. Hydrogen bonds are indicated with orange dotted lines.

Docking studies were performed to explore the binding mode of **2**–**4** within the binding site of hBuChE (PDB: 4TPK [30]), for which they showed nM inhibitory potencies. The three compounds gave binding poses consistent with carbamoylation of the catalytic residue S198, with the carbamate group close to the catalytic triad (S198, H438 and E325) and forming hydrogen bonds with the oxyanion-hole residues (G116 and G117). The compounds adopt a folded conformation, with the propylaminomethyl linker and the coumarin group occupying the entrance of the gorge. The carbamate O-phenyl ring is accommodated at the bottom of the binding site, delimited by the indole nucleus of W82. The amino group, modelled in its protonated state, takes polar contacts with the hydroxyl group of T120. The binding of the three compounds is stabilized by different polar interactions between hydrophilic residues situated at the entrance of the gorge and the coumarin portion. The acyl chain binding pocket of hBuChE, delimited by L286 and V288, contains the carbamate N-substituent. In particular, the naphthyl nucleus of **4** is well accommodated in this pocket, occupying the same region as the naphthamide moiety of a selective, non-covalent BuChE inhibitor co-crystallized within the X-ray structure 4TPK (Figure 3A). Notably, this hydrophobic cleft is characterized by a reduced volume in hAChE, with L286 and V288 of hBuChE replaced by the bulkier residues F295 and F297, which hamper the accommodation of large N-substituents. The reduced volume can account for the selectivity observed for the new inhibitors, with a 100-fold difference in IC_50_ values observed for the naphthyl derivative **4**. When docked in the substrate binding site of hAChE (PDB: 4EY6 [31]), this compound is not able to properly insert the carbamate group in the catalytic site due to steric clashes of the naphthyl ring with the two phenylalanines and the non-optimal accommodation of the inhibitor is likely reflected in the lower inhibitory potency toward hAChE (Figure 3B).

**Figure 4:**
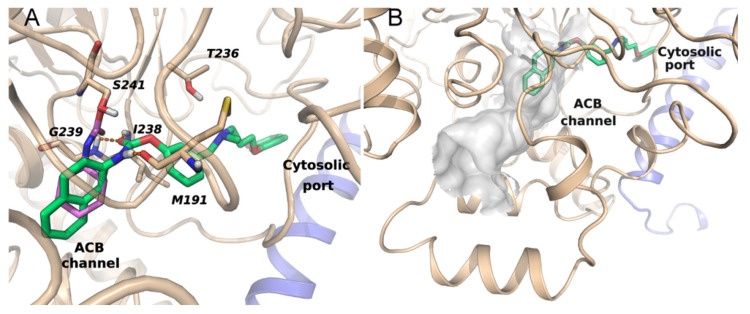
(**A**) Binding mode of **4** (green carbons) within the active site of rFAAH. The coumarin ring occupies the cytosolic port at the interface between the two subunits of the FAAH homodimer (chain A, light blue ribbons; chain B, beige ribbons). The *N*-naphthyl substituent fits the ACB channel and overlaps the cyclohexyl fragment of URB597 (purple carbons) covalently bound to the catalytic serine in the X-ray structure 3LJ7, superposed to the docking complex. Polar interactions are marked with orange dotted lines. (**B**) Surface of the ACB channel of FAAH with docked **4**.

Docking studies were also performed to investigate the binding mode of compounds **2**–**4** within the active site of rat FAAH (PDB: 1MT5 [32]). We started from the hypothesis, supported by the lack of activity of amide analogues **5–7** and the increase in potency observed with inhibitor pre-incubation (Table 2), that these compounds carbamoylate the active serine, similarly to what had been proposed for compounds of the same series [20]. We thus analyzed the induced-fit-docking poses that accommodate the carbamate carbonyl within the oxyanion site of the enzyme. The coumarin nucleus and the propylaminomethyl linker occupy a wide region situated at the interface between the two subunits of the FAAH homodimer. This region, also referred to as the cytosolic port, is lined by hydrophilic residues and represents the exit route of the ethanolamine released upon cleavage of the substrate anandamide. More specifically, **2**–**4** occupy the upper region of the cytosolic port cleft, similar to the arrangement seen for other carbamate FAAH inhibitors having the same O-substituent. The cyclic N-substituents are positioned in the hydrophobic acyl chain binding (ACB) channel, which is involved in the recruitment of the substrate from the membrane, and is occupied, in a crystallized structure (PDB: 3LJ7 [33]), by the cyclohexyl fragment of the reference FAAH inhibitor URB597 covalently bound to S241 (Figure 4A). The cycloalkyl carbamates **2** and **3** showed lower FAAH inhibitory potencies than previously described analogues with primary alkyl chains at the nitrogen atom. This can be ascribed to the greater steric bulk, which influence the overall accommodation of the inhibitor. In fact, the docking poses of **2** and **3** do not allow the formation of a hydrogen bond between the methylamino group of the ligands and T236, contrary to what had been observed for primary N-alkyl derivatives. On the other hand, the naphthyl nucleus is better tolerated at FAAH binding site, with a partial recovery of inhibitory potency. This apparently contrasts with the lack of activity observed for the *p*-hexylphenyl-carbamate in a previous work [20]. Actually, the docking pose (Figure 4A) shows that the naphthyl nucleus is accommodated within the first portion of the ACB pocket, similarly to the *N*-cyclohexyl group of other covalent inhibitors (Figure 4B). As the ACB channel makes a turn just after this first portion of space, bulky substituents at the para position of a phenyl ring would cause steric clashes. 

#### 2.2.4. CB Receptors Binding Assay for Compounds **5**–**7**

Taking advantage of the widespread placement of the amide function in potent CB ligands, the ability of the newly synthesized compounds **5–7** to bind these receptors was also evaluated. The binding affinities of compounds **5–7** at human recombinant CB1 and CB2 receptors were determined by a competition assay using a high affinity radioligand, i.e., [^3^H]-CP-55,940 and results are reported in Table 4.

Derivatives **5**–**7** appear weak ligands, with IC_50_/Ki in the μM range, showing compounds **5** and **6** the highest potency on CB2 and compound **7** a slight selectivity for CB1. 

#### 2.2.5. Inhibition of Aβ_42_ Self-Aggregation for Compounds **2**–**7**

Finally, in a multitarget perspective, the compounds were tested to evaluate their A*β* anti-aggregation properties. Indeed, FAAH proved to co-localize with Aβ rich plaques and activated astrocytes, suggesting a correlation between FAAH expression and AD-related neuropathologic changes [34]. The results indicate a weak inhibitory activity on self-Aβ aggregation, allowing some interesting considerations: the nature of the terminal function (carbamic or amidic) appears irrelevant for activity (**2** vs. **6** and **3** vs. **7**), whereas the *N*-substituent seems to play a more prominent role, being the cycloaliphatic groups more effective than the naphthyl one in the carbamate series and the cyclopentyl more than the cyclohexyl one in the amide series. 

## 3. Discussion

Starting from a previously reported lead compound **1**, suitably designed modifications were applied in a multitarget perspective, with a particular focus on the carbamate moiety. Besides, in a small number of compounds the carbamate was replaced with an amide group, widely reported in medicinal chemistry. The new derivatives were evaluated for their activities on a panel of the selected target embroiled in AD. The results proved that the carbamic derivatives **2** and **3** were endowed with promising inhibitory activities on AChE, BuChE, FAAH and Aβ_42_ self-aggregation, combined with calculated brain-to-blood ratios lower than rivastigmine (QPLogBB in Table 1), but better than the *n-*heptyl lead **1**. Undoubtedly, the potencies related to the single activities of these twocompounds appear moderate, but, remarkably, all the selected targets are involved in the pathogenesis and progression of the same disease and, from a multitarget point of view, this behaviour acquires a pivotal relevance. Indeed, the derivatives may counteract AD by simultaneously modulating different networked pathways involved in the disease development. The cholinergic system and ACh play a crucial role in learning and in the formation of memory. A vicious circle is recognized between Aβ and AChE: AChE directly interacts with soluble Aβ and facilitates its precipitation around plaques, Aβ induce cholinergic system dysfunction and a severe loss of AChE. Additionally, an excess of Aβ can simultaneously affect AEA production and degradation, by both reducing the availability of its biosynthetic precursor NArPE and by overexpressing FAAH, respectively [35]. 

It should be noted that levels of BuChE, the enzyme that compensates for AChE in hydrolyzing central ACh, increase as the disease progresses. Moreover, recent studies demonstrate that BuChE can hydrolyze 2-AG to arachidonic acid, which may give evidence for a more specific role of this enzyme in endocannabinoid regulation by CB receptors activation [36]. This action will be added to the inhibition of endocannabinoid inactivation by FAAH, obtaining beneficial effects in terms of both anti-inflammatory and neuroprotective effects and, subsequently, anti-amnesic actions. On the other hand, activation of CB1 was suggested to contribute to the amnesic actions of Aβ or to counteract these actions, depending on the timing of administration, and to inhibit Aβ toxicity in vitro. CB2 levels increase in AD and its stimulation counteracts microglia activation induced by Aβ, a beneficial effect found in both in vitro and in vivo models and stimulates Aβ removal by macrophages.

In the amide series, compound **6** emerged as an interesting multipotent inhibitor of AChE, BuChE and Aβ_42_ self-aggregation, as well as a potential CB1/CB2 receptors ligand.

In summary, some of these compounds show an encouraging multitarget profile and deserve further pharmacological characterization to deeper exploit their therapeutic potential.

## 4. Materials and Methods

### 4.1. Chemistry

#### 4.1.1. General Methods

Starting materials, unless otherwise specified, were used as high grade commercial products. Solvents were of analytical grade. Reaction progress was followed by thin layer chromatography (TLC) on precoated silica gel plates (Silica Gel 60 F254, Merck, Darmstadt, Germany and then visualized with a UV254 lamplight. Melting points were measured in glass capillary tubes on a SMP-20 apparatus (Büchi Italia, Assago, MI, Italy ) and are uncorrected. ^1^H-NMR and ^13^C-NMR experiments were recorded in CDCl_3_, unless differently indicated, on a VXR 400 MHz instrument (Varian, Palo Alto, CA). Chemical shifts are reported in parts per million (ppm) relative to tetramethylsilane (TMS), and spin multiplicities are given as s (singlet), d (doublet), t (triplet), m (multiplet) or br (broad). Direct infusion ESI-MS spectra were recorded on a Micromass ZQ 4000 apparatus (Waters, San Diego, CA, USA). Chromatographic separations were performed on silica gel columns (Kieselgel 40, 0.040−0.063 mm; Merck) by flash chromatography. Compounds were named relying on the naming algorithm developed by CambridgeSoft Corporation (Waltham, MA, USA) and used in ChemDraw Professional 15.0 (PerkinElmer Inc., Waltham, MA, USA).

#### 4.1.2. General Method for the Preparation of Carbamates **2–4**

A mixture of 7-(3-((3-hydroxybenzyl)(methyl)amino)propoxy)-2*H*-chromen-2-one (8) (1.0 mmol), the selected isocyanate (1.0 mmol) and NaH (10 mg) in dry toluene (20 mL) was stirred at rt for 24 h, quenched with water and then extracted with dichloromethane (3 × 20 mL). The organic layer was washed with water, dried and evaporated under reduced pressure. The residue was purified by flash column chromatography (toluene/acetone 3:2).

##### 3-((Methyl(3-((2-oxo-2H-chromen-7-yl)oxy)propyl)amino)methyl)phenyl cyclopentylcarbamate (**2**)

Using the previous procedure and starting from **8** and cyclopentane isocyanate compound **2** was obtained. Yield 30 %, oil. ^1^H-NMR δ 7.60 (d, *J* = 9.6 Hz, 1H), 7.31 (d, *J* = 9.2 Hz, 1H), 7.24–7.19 (m, 1H), 7.07 (t, *J* = 7.6 Hz, 2H), 6.94 (d, *J* = 8.0 Hz, 1H), 6.77 (d, *J* = 7.8 Hz, 2H), 6.21 (d, *J* = 9.6 Hz, 1H), 5.13 (br, 1H, NH), 4.02 (t, *J* = 6.3 Hz, 2H, OCH_2_), 3.47 (s, 2H, NCH_2_-Ph), 2.50 (t, *J* = 6.4 Hz, 2H, NCH_2_), 2.22 (s, 3H, NCH_3_), 2.01–1.93 (m, 5H), 1.69–1.62 (m, 2H), 1.60–1.51 (m, 2H), 1.49–1.46 (m, 2H). ^13^C-NMR δ 162.5, 161.6, 155.7, 153.8, 151.3, 143.2, 140.4, 128.8, 126.0, 122.3, 120.4, 116.7, 113.5, 113.0, 112.6, 101.2, 66.6, 62.4, 55.2, 52.2, 42.8, 33.9, 33.6, 27.1, 25.6, 25.4. ESI-MS *m/z*: 451 (M + H^+^).

##### 3-((Methyl(3-((2-oxo-2H-chromen-7-yl)oxy)propyl)amino)methyl)phenyl cyclohexylcarbamate (**3**)

Using the previous procedure and starting from **8** and cyclohexyl isocyanate, compound **3** was obtained. Yield 80 %, oil. ^1^H-NMR δ 7.64 (d, *J =* 9.2 Hz, 1H), 7.35 (d, *J =* 8.8 Hz, 1H), 7.27–7.24 (m, 1H), 7.12 (d, *J =* 9.2 Hz, 2H), 7.00 (d, *J =* 8.0 Hz, 1H), 6.80 (d, *J =* 6.8 Hz, 2H), 6.23 (d, *J =* 9.6, 1H), 5.04 (br, 1H, NH), 4.07 (t, *J =* 6.4 Hz, 2H), 3.55 (s, 2H), 2.53 (t, *J =* 6.4 Hz, 2H), 2.29 (s, 3H), 2.18–1.93 (m, 4H), 1.76–1.72 (m, 3H), 1.64–1.61 (m, 2H), 1.43–1.32 (m, 4H). ^13^C-NMR δ 162.4, 161.4, 155.8, 153.8, 151.1, 143.6, 140.4, 128.6, 125.6, 122.1, 120.3, 116.2, 113.1, 112.9, 112.8, 101.3, 66.7, 62.0, 53.2, 50.2, 42.3, 33.9, 33.2, 26.8, 25.6, 25.4, 24.8. ESI-MS *m/z*: 465 (M + H^+^)

##### 3-((Methyl(3-((2-oxo-2H-chromen-7-yl)oxy)propyl)amino)methyl)phenyl naphthalen-2-ylcarbamate (**4**)

Using the previous procedure and starting from **8** and 2-isocyanatonaphthalene, compound **4** was obtained. Yield 50 %, mp 190–192 °C. ^1^H-NMR δ 7.83–7.79 (m, 4H), 7.55 (d, *J =* 9.6 Hz, 1H), 7.49–7.45 (m, 2H), 7.31–7.25 (m, 2H), 7.18 (d, *J =* 8.4 Hz, 2H), 7.11 (d, *J =* 7.6 Hz, 1H), 7.04 (d, *J* = 7.6, 1H), 6.81–6.79 (m, 2H), 6.19 (d, *J =* 9.6 Hz, 1H), 5.81 (br, 1H, NH), 4.04 (t, *J =* 6.8 Hz, 2H), 3.51 (s, 2H), 2.52 (t, *J =* 6.8 Hz, 2H), 2.28 (s, 3H), 2.01–1.94 (m, 2H). ^13^C-NMR δ 161.4, 155.8, 154.4, 151.7, 143.4, 135.3, 133.5, 133.0, 130.6, 129.1, 128.8, 128.3, 127.9, 127.8, 126.5, 126.4, 126.2, 126.1, 125.9, 124.7, 123.7, 113.6, 113.2, 112.1, 102.2, 65.4, 59.3, 52.6, 40.0, 24.2. ESI-MS *m/z*: 509 (M + H^+^).

#### 4.1.3. General Method for the Preparation of Acetamides **5**–**7**

To a solution of 8 (1.0 mmol) in acetone (30 mL), the selected iodoacetamide (12–14, 1.0 mmol) and K_2_CO_3_ were added and the mixture was stirred and refluxed for 24 h. Upon reaction completion, the mixture was hot filtered and the solvent was evaporated under reduced pressure. The resulting crude product was purified by flash column chromatography (toluene/acetone 4:1), and then triturated with petroleum ether.

##### N-Heptyl-2-(3-((methyl(3-((2-oxo-2H-chromen-7-yl)oxy)propyl)amino)methyl)phenoxy)acetamide (**5**)

Using the previous procedure and starting from **8** and *N*-heptyl-2-iodoacetamide (**12**), the final acetamide **5** was obtained. Yield 30 %, mp 40–42 °C. ^1^H-NMR δ 7.63 (d, *J =* 9.6 Hz, 1H), 7.36 (d, *J =* 8.4 Hz, 1H), 7.27–7.21 (m, 1H), 6.96 (d, *J =* 7.2 Hz, 1H), 6.91 (s, 1H), 6.82–6.77 (m, 3H), 6.81 (br, 1H), 6.25 (d, *J =* 9.6 Hz, 1H), 4.44 (s, 2H), 4.09 (t, *J =* 6.4 Hz, 2H), 3.50 (s, 2H), 3.35–3.30 (m, 2H), 2.56 (t, *J =* 6.8 Hz, 2H), 2.25 (s, 3H), 2.03–1.98 (m, 2H), 1.55–1.50 (m, 2H), 1.34–1.26 (m, 10H), 0.88 (t, *J =* 6.8 Hz, 3H). ^13^C-NMR δ 162.6, 161.7, 156.4, 156.0, 143.8, 140.4, 129.5, 128.8, 121.2, 116.2, 114.6, 113.3, 112.9, 112.5, 101.5, 67.5, 66.7, 53.6, 42.4, 39.2, 31.8, 29.7, 29.1, 26.9, 22.7, 14.2, 11.3. ESI-MS *m/z*: 495 (M + H^+^).

##### N-Cyclopentyl-2-(3-((methyl(3-((2-oxo-2H-chromen-7yl)oxy)propyl)amino)methyl)phenoxy)-acetamide (**6**)

Using the previous procedure and starting from **8** and N-cyclopentyl-2-iodoacetamide (**13**), the final acetamide **6** was obtained. Yield 45 %, oil. ^1^H-NMR δ 7.65 (d, J = 9.6 Hz, 1H), 7.36 (d, J = 8.4 Hz, 1H), 7.15 (t, J = 7.8 Hz, 1H), 6.91 (s, 1H), 6.88 (s, 1H), 6.84–6.80 (m, 2H), 6.75–6.73 (m, 1H), 6.26 (d, J = 9.2 Hz, 1H), 4.11 (t, J = 6.2 Hz, 2H), 3.48 (s, 2H), 2.57 (t, J = 6.4 Hz, 2H), 2.25 (s, 3H), 2.03–1.98 (m, 3H), 1.65–1.58 (m, 4H), 1.44–1.36 (m, 4H). ^13^C-NMR δ 162.6, 161.7, 156.4, 156.0, 143.8, 140.4, 129.5, 128.8, 121.2, 116.2, 114.6, 113.3, 112.9, 112.5, 101.5, 66.9, 62.5, 54.8, 53.6, 42.2, 42.3, 33.2, 27.2, 26.9, 23.8, 23.7. ESI-MS *m/z*: 465 (M + H^+^).

##### N-Cyclohexyl-2-(3-((methyl(3-((2-oxo-2H-chromen-7-yl)oxy)propyl)amino)methyl)phenoxy)-acetamide (**7**)

Using the previous procedure and starting from **8** and *N*-cyclohexyl-2-iodoacetamide **14**, the final acetamide **7** was obtained. Yield 25 %, oil. ^1^H-NMR δ 7.63 (d, *J* = 9.6 Hz, 1H), 7.35 (d, *J* = 7.6 Hz, 1H), 7.22 (t, *J* = 8 Hz, 1H), 6.95 (d, *J* = 7.2 Hz, 1H), 6.91 (s, 1H), 6.80–6.78 (m, 3H), 6.42 (d, *J* = 8 Hz, 1H), 6.24 (d, *J* = 9.5 Hz, 1H), 4.42 (s, 2H), 4.08 (t, *J* = 6 Hz, 2H), 3.86–3.84 (m, 1H), 3.50 (s, 2H), 2.58 (t, *J* = 6.9 Hz, 2H), 2.25 (s, 3H), 2.03–2.00 (m, 2H), 1.92–1.90 (m, 2H), 1.68–1.61 (m, 4H), 1.41–1.32 (m, 2H), 1.25–1.16 (m, 2H). ^13^C-NMR δ 167.3, 162.3, 161.4, 157.4, 155.9, 143.6, 141.1, 129.6, 128.8, 122.7, 115.3, 113.4, 113.1, 112.9, 112.5, 101.6, 67.4, 66.7, 62.3, 53.6, 47.9, 42.3, 33.2, 33.1, 26.9, 25.5, 25.0, 24.9. ESI-MS m/z: 479 (M + H^+^).

#### 4.1.4. General Method for the Preparation of Amide Derivatives **9–11**

To a solution of selected amine (4.34 mmol) in acetone (20 mL), anhydrous K_2_CO_3_ (1.0 g) and 2-chloroacetyl chloride (4.34 mmol) were added and the mixture was stirred and refluxed for 24 h. Upon reaction completion, the mixture was hot filtered and the solvent was evaporated under reduced pressure. The residue was purified by flash column chromatography (petroleum ether/ethyl acetate 4:1). The compounds are already reported in literature with different synthetic procedures. The full characterization can be found in the corresponding reference. 

##### 2-Chloro-N-heptylacetamide (**9**)

Using the previous procedure and starting from heptylamine, compound **9** was obtained [37]. Yield 89 %, oil. ^1^H-NMR δ 6.65 (br, 1H), 4.02 (s, 2H), 3.30–3.25 (m, 2H), 1.56–1.49 (m, 2H), 1.32–1.28 (m, 8H), 0.88 (t, *J* = 7.2 Hz, 3H) 

##### 2-Chloro-N-cyclopentylacetamide (**10**)

Using the previous procedure and starting from cyclopentylamine, compound **10** was obtained [38]. Yield 95 %, oil. ^1^H-NMR δ 6.47 (br, 1H), 4.25–4.20 (m, 1H), 4.02 (s, 2H), 2.06–1.98 (m, 2H), 1.75–1.62 (m, 4H), 1.48–1.40 (m, 2H).

##### 2-Chloro-N-cyclohexylacetamide (**11**)

Using the previous procedure and starting from cyclohexylamine, compound **11** was obtained [39]. Yield 85 %, oil. ^1^H-NMR δ 6.51 (br, 1H), 4.03 (s, 2H), 3.54–3.41 (m, 1H), 1.94–1.88 (m, 2H), 1.46–1.41 (m, 4H), 1.21–1.11 (m, 4H).

#### 4.1.5. General Method for the Preparation of Iodo Derivatives **12**–**14**

A mixture of the selected chloro derivative (1.0 mmol) and NaI (1.0 mmol) in methylethylketone (15 mL) was refluxed for 3 h, then it was concentrated under reduced pressure. The crude was dissolved in dichloromethane and washed with water. The organic layer was evaporated under reduced pressure, affording compounds **12–14** that were used for the next reaction without any further purification.

#### 4.1.6. General Method for the Preparation of Isocyanates

A mixture of selected benzoic acid (1.0 eq) and SOCl_2_ (10 mL) was refluxed for 5 h, then SOCl_2_ was removed. To an ice-cold mixture of NaN_3_ (1.4eq) previously dissolved in water (2 mL), a solution of the selected benzoyl chloride in acetone (2 mL) was added, keeping the temperature below 10 °C. The mixture was stirred for 1 h, then the layers were separated and the lower aqueous layer was discarded; the upper layer was slowly and dropwise added to 30 mL of benzene heated to 60 °C and the mixture was kept at 60–70 °C until gas production ceased; then it was filtered and the solvent was removed. The obtained oil was used as a crude, without any further purification, for next step of reaction.

### 4.2. Biological Evaluation Methods

#### 4.2.1. Determination of the Inhibitory Potency towards Human AChE and BuChE

The capacity of 2–7 to inhibit human ChE activity was assessed using the Ellman’s method [25]. Initial rate assays were performed at 37 °C with a V-530 double beam spectrophotometer (Jasco Corporation, Cremella (LC), Italy city, state/prov abbrev if USA/Canada, country). The rate of increase in the absorbance at 412 nm was followed for 180 s. AChE stock solution was prepared by dissolving human recombinant AChE lyophilized powder (Sigma-Aldrich, Merck group, Milan, Italy) in 0.1 M phosphate buffer at pH 8.0 containing 0.1% Triton X-100. Stock solution of BuChE was prepared by diluting commercial stock solution of BuChE from human serum (Sigma) in an aqueous solution of gelatin (0.2%) in order to have a final activity comprised between 0.11–0.15 ΔAbs/min. Stock solutions of inhibitors (1–2 mM) were prepared in methanol and diluted in methanol. For IC_50_ assessment, five/six increasing concentrations of the inhibitor were evaluated, able to give an inhibition of the enzymatic activity in the range of 20–80%. The assay solution consisted of a 0.1 M phosphate buffer pH 8.0, with the addition of 340 µM (Ellman’s reagent, 0.02 unit/mL of human recombinant AChE or BuChE from human serum and 550 µM of substrate (acetylthiocholine iodide-ATCh or butyrylthiocholine iodide–BTCh for AChE and BuChE, respectively). Aliquots of the tested inhibitor at increasing concentration (or methanol) were added to the assay solution and incubated with the enzyme at 37 °C for 20 min for non-covalent derivatives 5–7, and for the time required to reach a stable inhibition value (carbamoylation plateau) for carbamates 2–4, before the addition of the substrate. Incubation times were: 60 min for 2 and 90 min for 3 and 4 on hAChE assay, and 45 min for 2 and 60 min for 3 and 4 on hBuChE assay, as assessed on the preliminary determination of the carbamoylation rate. Blank solutions containing all components except the enzyme were prepared in order to account for the non-enzymatic hydrolysis of the substrate. The reaction rates obtained in the absence and presence of inhibitor were compared and the percent inhibition due to the presence of inhibitor was calculated. Each concentration was analyzed in duplicate/triplicate, and IC_50_ values were determined graphically from log concentration–inhibition curves (GraphPad Prism 4.03 software, GraphPad Software Inc., San Diego, CA). Two independent experiments were performed for the determination of each IC_50_ value or for the calculation of the % of inhibition where the IC_50_ value could not be determined because of low inhibitory activity. 

#### 4.2.2. Determination of Carbamoylation Events for Carbamic Derivatives **2**–**4**

A stopped time assay was performed in order to define the time required for enzyme carbamoylation. In detail, residual enzyme activity upon selected incubation times was assessed by following the same protocol described in section “Determination of the inhibitory potency towards human AChE and BuChE”. Briefly, the specific cholinesterase enzyme (either hAChE or hBuChE) was mixed in the assay buffer at pH 8.0 in the presence of DTNB (340 µM final concentration) with a concentration of inhibitor equal to its IC_50_ value. The mixture was incubated at 37 °C for given times (from 5 to 90 min) before the addition of the substrate (AChT or BChT, respectively) and the determination of residual enzyme activity by Ellman’s method. A parallel control, with no inhibitor, allowed to adjust activities measured at various time. 

#### 4.2.3. Inhibition of Aβ_42_ Self-Aggregation

As reported in a previously published protocol [40], 1,1,1,3,3,3-hexafluoro, 2-propanol pretreated Aβ_42_ samples (Bachem AG, Bubendorf, Switzerland) were solubilized with a CH_3_CN/0.3 mM Na_2_CO_3_/250 mM NaOH (48.4:48.4:3.2) mixture. Experiments were performed by incubating the peptide in 10 mM phosphate buffer (pH = 8.0) containing 10 mM NaCl, at 30 °C for 24 h (final Aβ concentration = 50 μM) with and without inhibitor (50 μM, Aβ/inhibitor = 1/1). Blanks containing the tested inhibitors were also prepared and tested. To quantify amyloid fibrils formation, the thioflavin T (ThT) fluorescence method was used [41]. After incubation, samples were diluted to a final volume of 2.0 mL with 50 mM glycine-NaOH buffer (pH 8.5) containing 1.5 μM ThT. A 300 s-time scan of fluorescence intensity was carried out (λ_exc_ = 446 nm; λ_em_ = 490 nm, FP-6200 fluorometer, Jasco Europe), and values at plateau were averaged after subtracting the background fluorescence of 1.5 μM ThT solution. The fluorescence intensities were compared and the percent inhibition due to the presence of the inhibitor.

#### 4.2.4. FAAH Assay

The effect of compounds on FAAH activity was detected by coincubating them with the 10,000× *g* membrane fraction of rat brain (70 μg/sample) and synthetic N-arachidonoyl-[14C]-ethanolamine (110 mCi/mmol, ARC, St. Louis, MO, USA) properly diluted with AEA (TocrisBioscience, Avonmouth, Bristol, UK) in Tris−HCl 50 mM, at pH 9.00−10.00 at 37 °C for 30 min. For the preincubation protocol, compounds were preincubated at various concentrations in the presence of the enzyme (10,000× *g* membrane fraction of rat brain, 70 μg/sample) for 20 min at 37 °C. The reaction was then initiated by adding the substrate. After incubation, the amount of [14C]-ethanolamine produced was measured by scintillation counting of the aqueous phase after extraction of the incubation mixture with 2 volumes of CHCl3/MeOH 1:1 (by vol.). Data are expressed as means ± SD of at least three separate experiments of the concentration exerting 50% inhibition of [14C]-AEA hydrolysis (IC50) calculated by fitting sigmoidal concentration response curves by GraphPad.

#### 4.2.5. Competition Binding Assay

Competition binding assay for CB1 and CB2 receptors was performed as previously reported [42]. Briefly, membranes from HEK-293 cells over-expressing the respective human recombinant CB1R (Bmax = 2.5 pmol/mg protein) and human recombinant CB2R (Bmax = 4.7 pmol/mg protein) were incubated with [^3^H]-CP-55,940 (0.14 nM/Kd = 0.18 nM and 0.084 nM/Kd = 0.31 nM, respectively, for CB1R and CB2R) as the high affinity ligand. Competition curves were performed by displacing [^3^H]-CP-55,940 with increasing concentration of compounds (0.1 nM–10 μM). Nonspecific binding was defined by 10 µM of WIN55,212-2 as the heterologous competitor (Ki values 9.2 nM and 2.1 nM, respectively, for CB1R and CB2R). All compounds were tested following the procedure described by the manufacturer (Perkin Elmer, Milan, Italy). Displacement curves were generated by incubating drugs with [3H]-CP-55,940 for 90 min at 30 °C.

#### 4.2.6. Computational Studies

Lipophilicity (QPlogPo/w), solubility (QPlogS) and brain/blood partition coefficient (QPlogBB) were calculated with QikProp using the default settings. Molecular modelling studies were performed using the Schrodinger 2018-2 software suite [43,44,45,46]. Ligands were built using Maestro 11.6 [47] and prepared with LigPrep 4.2 [48]. Protein structures were prepared using the Protein Preparation Wizard tool. Induced Fit Docking (IFD) studies were conducted using Glide 7.9 applying the SP scoring function [49].

#### 4.2.7. Protein Preparation

Chains A and B of the X-ray structure of rat FAAH covalently bound to methylarachidonyl phosphonate (MAP; PDB 1MT5) were used to perform IFD calculations. The covalent inhibitor was removed, and missing hydrogen atoms were added. The overall hydrogen bonding network was optimized by adjusting histidine tautomers and by sampling the orientation of hydroxyl and thiol groups and the side chain amides of asparagine and glutamine residues. Acid and basic amino acids were modelled in their charged state, while K142, which is part of the catalytic triad of FAAH, was maintained neutral. The structure was submitted to a minimization performed with the OPLS2005 force field [50] during which only hydrogen atoms were allowed to move. A second minimization run was performed restraining the position of heavy atoms to an RMSD value of 0.3 Å from the X-ray structure. All the co-crystallized water molecules were removed before running docking studies.

Chain A of the crystallographic structure of hBuChE in complex with a non-covalent inhibitor (4TPK [30]) and chain B of hAChE in complex with galantamine (4EY6 [31]) were used to perform IFD studies. The proteins were prepared following the same procedure described above, removing the inhibitor and water molecules before docking studies.

#### 4.2.8. rFAAH Induced Fit Docking Studies

Compounds were docked in their neutral state, following a protocol previously applied to similar inhibitors [20].^.^In fact, coumarin analogues with the same propylaminomethyl linker had shown a significant loss of potency at pH 7.4, compared to pH 9.0, which supports the hypothesis that these derivatives bind FAAH in their neutral state [20]. The docking grid was built using Glide 7.9. In the first step of the IFD protocol, a softened-potential docking run was performed setting van der Waals scaling factors to 0.7 and 0.5 for the protein and ligand non-polar atoms, respectively, and the side chain of L278 was temporarily mutated to alanine. Hydrogen bond constraints between the carbamate group of the ligands and the backbone NH groups of I238 and G239, and the backbone oxygen atom of M191 were imposed to obtain complexes consistent with the carbamoylation-based mechanism of these compounds. The resulting complexes were thus submitted to a refinement process, in which the side chain of L278 was restored and the side chains of amino acids within 5 Å from the ligand were refined through a conformational search using Prime 5.2 [46]. The resulting complexes were minimized according to the Prime routine. In the last docking step, each ligand was optimized in the field of the receptor and scored with default Glide settings. The complexes were ranked according to their IFD score and the best-ranked complexes were energy-minimized applying the OPLS2005 force field implemented in MacroModel 12.0 [51] using the Polak-Ribiere conjugate gradient method to a convergence threshold of 0.05 kJ mol^−1^ Å^−1^. During minimization, only the ligands and the surrounding residues within 10 Å were free to move, while the backbone of other residues was kept fixed.

#### 4.2.9. hBuChE and hAChE Induced Fit Docking Studies

Docking studies were performed following the protocol described in [20]. Compounds were docked in their protonated state, given the ability of cholinesterases to accommodate positively charged groups. The docking grid was built using Glide 7.9. A preliminary softened-potential docking run was performed setting van der Waals radii scaling of protein and ligand non-polar atoms to 0.7 and 0.6, respectively. A positional constraint was applied on the carbamate group, to obtain complexes consistent with the carbamoylation-based mechanism of these compounds. The ligand-protein complexes were then submitted to a refinement procedure, in which the side chains of amino acids within 5 Å from the ligand, with the exception of S198, H438, E325, W82 for hBuChE and S203, H447, E334, W86 for hAChE, were refined through a conformational search. After minimization, in the final docking step each inhibitor was optimized in the field of the receptor and scored according to the default Glide settings. Finally, the complexes with best IFD score were energy-minimized applying the OPLS2005 force field implemented in MacroModel 12.0 using the Polak-Ribiere conjugate gradient method to a convergence threshold of 0.05 kJ mol^−1^ Å^−1^. During minimization, only the ligands and the surrounding residues within 10 Å were free to move, while the backbone of other residues was kept fixed.

## Figures and Tables

**Table 1:** Lipophilicity, solubility and blood-brain barrier permeation calculated for target compounds and rivastigmine.

Comp	QPlogPo/w *^a^*	QPlogS *^a^*	QPlogBB *^a,b^*
**1**	5.46	−7.55	−1.65
**2**	4.16	−5.74	−1.01
**3**	4.47	−6.17	−1.02
**4**	5.27	−6.94	−1.11
**Riva**	2.46	−2.25	0.41
**5**	4.89	−6.07	−1.72
**6**	3.85	−5.14	−1.22
**7**	4.15	−5.52	−1.22

*^a^* Calculated with QikProp (Schrodinger LLC); *^b^* Brain/blood partition coefficient.

**Table 2:** Inhibitory activities against hAChE, hBuChE, *r*FAAH (IC_50_), and Aβ_42_ self-aggregation (% inhibition) values of compounds **2–4** and of the marketed anti-AD drug Rivastigmine (Riva).

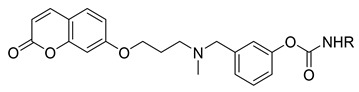						
Comp.	R	IC_50_ hAChE (±SEM) nM *^a^*	IC_50_ hBuChE (±SEM) nM *^a^*	IC_50_ FAAH (m ± sd) μM	IC_50_ FAAH with 20 min Pre-incubation μM	% Inhibition Aβ_42_ self Aggreg. *^b^* (±SEM)
**1**	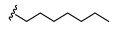	37.4 ± 1.9 *^c^*	1.36 ± 0.07 *^c^*	0.161 ± 0.027 *^c^*	0.029 ± 0.001 *^c^*	nd
**2**	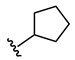	521 ± 14 *^d^*	8.42 ± 0.13 *^e^*	6.09 ± 1.55	2.53 ± 0.55	22.9 ± 1.3
**3**	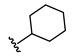	104 ± 21 *^f^*	34.9 ± 4.2 *^d^*	2.55 ± 0.33	1.33 ± 0.28	18.9 ± 2.1
**4**	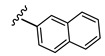	264 ± 0.05 *^f^*	2.00 ± 0.20 *^d^*	1.58 ± 0.26	0.63 ± 0.08	13.9 ± 3.8
**Riva**		1535 ± 64 *^g^*	301 ± 14 *^g^*	nd	nd	nd

*^a^* Human recombinant AChE and BuChE from human serum were used. IC50 values represent the concentration of inhibitor required to decrease enzyme activity by 50% and are the mean of two/three independent measurements, each performed in duplicate. SEM = standard error of the mean; *^b^* [Aβ42] = [inhibitor] = 50 µM; *^c^* Data from reference [20]; *^d^* Incubation time 60 min; *^e^* Incubation time 45 min; *^f^* Incubation time 90 min; *^g^* Data from reference [27]; nd stands for not determined.

**Table 3:** Inhibitory activity against *h*AChE, *h*BuChE, *r*FAAH and Aβ_42_ self-aggregation.

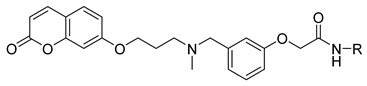					
Comp.	R	hAChE % Inhibition (±SEM) *^a^* [I] = 40 µM	hBuChE IC_50_ (±SEM) µM *^a^*	IC_50_ FAAH (m ± sd) μM	% Inhibition Aβ_42_ Self-Aggregation *^b^* (±SEM)
**5**	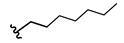	13.6 ± 5.1	182 ± 28	>1000	n.a.
**6**	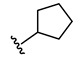	41.7 ± 0.5 *^c^*	11.6 ± 0.8	>1000	20.3 ± 1.9
**7**	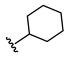	34.0 ± 3.2	12.8 ± 2.1	>1000	13.5 ± 5.0

*^a^* Human recombinant AChE and BuChE from human serum were used. IC_50_ values represent the concentration of inhibitor required to decrease enzyme activity by 50% and are the mean of two/three independent measurements, each performed in duplicate. SEM = standard error of the mean; *^b^* [Aβ42] = [inhibitor] = 50 µM; *^c^* IC_50_ = (59.4 ± 1.0) µM; na stands for not active, i.e., % inhibition lower than 10% at the tested concentration.

**Table 4:** Effect of compounds **5–7** on [^3^H]-CP55940 binding to the human recombinant CB1 and CB2 receptors.

Comp.	Ki on CB1 (m ± sd) µM	Max Tested on CB1 (% Displacement) (m ± sd) µM	Ki on CB2 (m ± sd) µM	Max Tested on CB2 (% Displacement) (m ± sd) µM
**5**	>10	10 (35.57 ± 0.43)	>10	10 (41.41 ± 0.87)
**6**	>10	10 (35.75 ± 0.65)	>10	10 (48.33 ± 1.73)
**7**	>10	10 (39.62 ± 10.48)	>10	10 (14.3 ±3.83)

## Sample Availability

Samples of the compounds are unavailable from the authors.

## References

[1] Alzheimer’s Association (2018). 2018 Alzheimer’s disease facts and figures. Alzheimers Dement..

[2] World Health Organisation (2017). Dementia Fact Sheet. http://www.who.int/news-room/fact-sheets/detail/dementia.

[3] Du X., Wang X., Geng M. (2018). Alzheimer’s disease hypothesis and related therapies. Transl. Neurodegener..

[4] Contestabile A. (2011). The history of the cholinergic hypothesis. Behav. Brain Res..

[5] Massoud F., Gauthier S. (2010). Update on the pharmacological treatment of Alzheimers disease. Curr. Neuropharmacol..

[6] Wang H., Zhang H. (2019). Reconsideration of Anticholinesterase Therapeutic Strategies against Alzheimer’s Disease. ACS Chem. Neurosci..

[7] Hampel H., Mesulam M.-M., Cuello A.C., Farlow M.R., Giacobini E., Grossberg G.T., Khachaturian A.S., Vergallo A., Cavedo E., Snyder P.J. (2018). The cholinergic system in the pathophysiology and treatment of Alzheimer’s disease. Brain.

[8] Hampel H., Mesulam M.-M., Cuello A.C., Khachaturian. A.S., Vergallo A., Farlow M.R., Snyder P.J., Giacobini E., Khachaturian Z.S. (2019). Revisiting the cholinergic hypothesis in Alzheimer’s Disease: Emerging evidence from translation and clinical research. J. Prev. Alzheimer Dis..

[9] Greig N.H., Utsuki T., Ingram D.K., Wang Y., Pepeu G., Scali C., Yu Q.S., Mamczarz J., Holloway H.W., Giordano T. (2005). Selective butyrylcholinesterase inhibition elevates brain acetylcholine, augments learning and lowers Alzheimer beta-amyloid peptide in rodent. Proc. Natl. Acad. Sci. USA.

[10] Pertwee R.G., Howlett A.C., Abood M.E., Alexander S.P., Di Marzo V., Elphick M.R., Greasley P.J., Hansen H.S., Kunos G., Mackie K. (2010). International union of basic and clinical pharmacology. LXXIX. Cannabinoid receptors and their ligands: Beyond CB1 and CB2. Pharmacol. Rev..

[11] Di Marzo V. (2006). Endocannabinoids: Synthesis and degradation. Rev. Physiol. Biochem. Pharmacol..

[12] Di Marzo V. (2008). Targeting the endocannabinoid system: To enhance or reduce?. Nat. Rev. Drug Discov..

[13] Fonseca B.M., Costa M.A., Almada M., Correia-da-Silva G., Teixeira N.A. (2013). Endogenous cannabinoids revisited: A biochemistry perspective. Prostaglandins Lipid Mediat..

[14] Micale V., Mazzola C., Drago F. (2007). Endocannabinoids and neurodegenerative diseases. Pharmacol. Res..

[15] Talarico G., Trebbastoni A., Bruno G., de Lena C. (2019). Modulation of the Cannabinoid System: A New Perspective for the Treatment of the Alzheimer’s Disease. Curr. Neuropharmacol..

[16] Ahn K., Johnson D.S., Cravatt B.F. (2009). Fatty acid amide hydrolase as a potential therapeutic target for the treatment of pain and CNS disorders. Expert Opin. Drug Discov..

[17] Cavalli A., Bolognesi M.L., Minarini A., Rosini M., Tumiatti V., Recanatini M., Melchiorre C. (2008). Multi-target-directed ligands to combat neurodegenerative diseases. J. Med. Chem..

[18] Morphy R., Rankovic Z. (2009). Designing multiple ligands–medicinal chemistry strategies and challenges. Curr. Pharm. Des..

[19] Rampa A., Bartolini M., Bisi A., Belluti F., Gobbi S., Andrisano V., Ligresti A., Di Marzo V. (2012). The first dual ChE/FAAH inhibitors: New perspective for Alzheimer’s disease?. ACS Med. Chem. Lett..

[20] Montanari S., Scalvini L., Bartolini M., Belluti F., Gobbi S., Andrisano V., Ligresti A., Di Marzo V., Rivara S., Mor M. (2016). Fatty Acid Amide Hydrolase (FAAH), Acetylcholinesterase (AChE), and Butyrylcholinesterase (BuChE): Networked Targets for the Development of Carbamates as Potential Anti-Alzheimer’s Disease Agents. J. Med. Chem..

[21] Supuran C.T. (2020). Coumarin carbonic anhydrase inhibitors from natural sources. J. Enzym. Inhib. Med. Chem..

[22] Menezes J., Diederich M. (2019). Translational role of natural coumarins and their derivatives as anticancer agents. Fut. Med. Chem..

[23] Mor M., Rivara S., Lodola A., Plazzi P.V., Tarzia G., Duranti A., Tontini A., Piersanti G., Kathuria S., Piomelli D. (2004). Cyclohexylcarbamic acid 3′- or 4′-substituted biphenyl-3-yl esters as fatty acid amide hydrolase inhibitors: Synthesis, quantitative structure-activity relationships, and molecular modeling studies. J. Med. Chem..

[24] Kumari S., Carmona A.V., Tiwari A.K., Trippier P.C. (2020). Amide Bond Bioisosteres: Strategies, Synthesis, and Successes. J. Med. Chem..

[25] Ellman G.L., Courtney K.D., Andres V., Featherstone R.M. (1961). A new rapid colorimetric determination of acetylcholinesterase activity. Biochem. Pharmacol..

[26] Main A.R., Hastings F.L. (1966). Carbamylation and Binding Constants for the Inhibition of Acetylcholinesterase by Physostigmine. Science.

[27] Bolognesi M.L., Bartolini M., Cavalli A., Andrisano V., Rosini M., Minarini A., Melchiorre C. (2004). Design, synthesis, and biological evaluation of conformationally restricted rivastigmine analogues. J. Med. Chem..

[28] Zha X., Lamba D., Zhang L., Lou Y., Xu C., Kang D., Chen L., Xu Y., Zhang L., De Simone A. (2016). Novel Tacrine–Benzofuran Hybrids as Potent Multitarget-Directed Ligands for the Treatment of Alzheimer’s Disease: Design, Synthesis, Biological Evaluation, and X-ray Crystallography. J. Med. Chem..

[29] Tuo W., Leleu-Chavain N., Spencer J., Sansook S., Millet R., Chavatte P. (2017). Therapeutic Potential of Fatty Acid Amide Hydrolase, Monoacylglycerol Lipase, and N-Acylethanolamine Acid Amidase Inhibitors. J. Med. Chem..

[30] Brus B., Košak U., Turk S., Pišlar A., Coquelle N., Kos J., Stojan J., Colletier J.P., Gobec S. (2014). Discovery, biological evaluation, and crystal structure of a novel nanomolar selective butyrylcholinesterase inhibitor. J. Med. Chem..

[31] Cheung J., Rudolph M.J., Burshteyn F., Cassidy M.S., Gary E.N., Love J., Franklin M.C., Height J.J. (2012). Structures of human acetylcholinesterase in complex with pharmacologically important ligands. J. Med. Chem..

[32] Bracey M.H., Hanson M.A., Masuda K.R., Stevens R.C., Cravatt B.F. (2002). Structural adaptations in a membrane enzyme that terminates endocannabinoid signaling. Science.

[33] Mileni M., Kamtekar S., Wood D.C., Benson T.E., Cravatt B.F., Stevens R.C. (2010). Crystal structure of fatty acid amide hydrolase bound to the carbamate inhibitor URB597: Discovery of a deacylating water molecule and insight into enzyme inactivation. J. Mol. Biol..

[34] Benito C., Nunez E., Tolon R.M., Carrier E.J., Rabano A., Hillard C.J., Romero J. (2003). Cannabinoid CB2 receptors and fatty acid amide hydrolase are selectively overexpressed in neuritic plaque-associated glia in Alzheimer’s disease brains. J. Neurosci..

[35] Jung K.-M., Astarita G., Yasar S., Vasilevko V., Cribbs D.H., Head E., Cotman C.W., Piomelli D. (2012). An amyloid β_42_-dependent deficit in anandamide mobilization is associated with cognitive dysfunction in Alzheimer’s disease. Neurobiol. Aging.

[36] Barricklow J., Blatnik M. (2013). 2-Arachidonoylglycerol is a substrate for butyrylcholinesterase: A potential mechanism for extracellular endocannabinoid regulation. Arch. Biochem. Biophys..

[37] Schweda S.I., Alder A., Gilberger T., Kunick C. (2020). 4-Arylthieno[2,3-b]pyridine-2-carboxamides Are a New Class of Antiplasmodial Agents. Molecules.

[38] Jöst C., Nitsche C., Scholz T., Roux L., Klein C.D. (2014). Promiscuity and Selectivity in Covalent Enzyme Inhibition: A Systematic Study of Electrophilic Fragments. J. Med. Chem..

[39] Cho S.-D., Song S.-Y., Park Y.-D., Kim J.-J., Joo W.-H., Shiro M., Falck J.R., Shin D.-S., Yoon Y.-J. (2003). Novel synthesis of pyridazino[4,5-b][1,4]oxazin-3,8-diones. Tetrahedron Lett..

[40] Bartolini M., Bertucci C., Bolognesi M.L., Cavalli A., Melchiorre C., Andrisano V. (2007). Insight into the kinetic of amyloid beta (1-42) peptide self-aggregation: Elucidation of inhibitors’ mechanism of action. ChemBioChem.

[41] Naiki H., Higuchi K., Nakakuki K., Takeda T. (1991). Kinetic analysis of amyloid fibril polymerization in vitro. Lab. Investig..

[42] Mugnaini C., Kostrzewa M., Bryk M., Mahmoud A.M., Brizzi A., Lamponi S., Giorgi G., Ferlenghi F., Vacondio F., Maccioni P. (2020). Design, Synthesis, and Physicochemical and Pharmacological Profiling of 7-Hydroxy-5-oxopyrazolo[4,3-b]pyridine-6-carboxamide Derivatives with Antiosteoarthritic Activity In Vivo. J. Med. Chem..

[43] Schrödinger Suite 2018-2 Protein Preparation Wizard. https://www.schrodinger.com/products/protein-preparation-wizard.

[44] (2018). Epik.

[45] (2018). Impact.

[46] (2018). Prime.

[47] (2018). Maestro.

[48] (2018). LigPrep.

[49] (2018). Glide.

[50] Jorgensen W.L., Maxwell D.S., Tirado-Rives J. (1996). Development and testing of the OPLS all-atom force field on conformational energetics and properties of organic liquids. J. Am. Chem. Soc..

[51] (2018). Macromodel.

